# 2,6-Diamino­pyridinium tetra­phenyl­borate–1,2-bis­(5,7-dimethyl-1,8-naphthyridin-2-yl)diazene (1/1)

**DOI:** 10.1107/S1600536811012943

**Published:** 2011-04-29

**Authors:** Bhanu P. Mudraboyina, Hong-Bo Wang, Roaxanne Newbury, James A. Wisner

**Affiliations:** a Department of Chemistry, The University of Western Ontario, Chemistry Building, 1151 Richmond Street, London, ON, Canada N6A 5B7

## Abstract

In the title compound, C_5_H_8_N_3_^+^·C_24_H_20_B^−^·C_20_H_18_N_6_, the 1,2-bis­(5,7-dimethyl-1,8-naphthyridin-2-yl)diazene mol­ecule is essentially planar (r.m.s. deviation = 0.0045 Å) and aligned in nearly coplanar manner with the 2,6-diamino­pyridinium ion, making a dihedral angle of 5.19 (5)°. The diamino­pyridine mol­ecule is protonated on the central pyridine N atom and the B atom bears the counter-charge. The amine groups of the diamino pyridinium cation form intra­molecular N—H⋯N hydrogen bonds, resulting in linear and bent inter­actions with the naphthyridine ring system.

## Related literature

For related literature, see: Blight *et al.* (2009 ▶); Li *et al.* (2010 ▶); Raboisson *et al.* (2007 ▶); Roma *et al.* (2010 ▶); Sahoo *et al.* (2010 ▶).
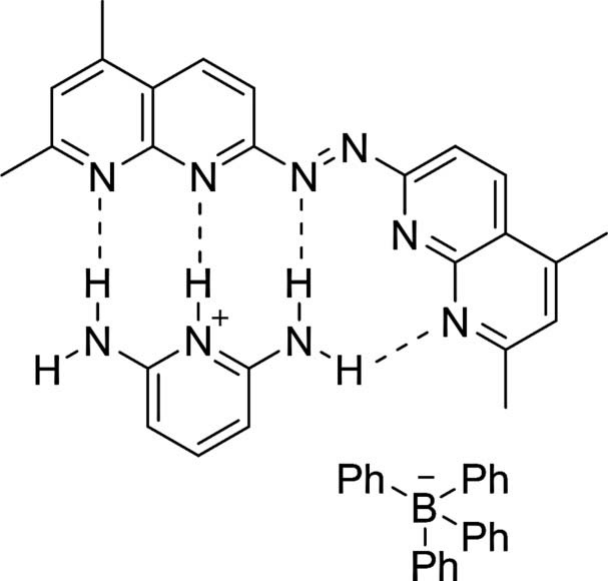


## Experimental

### 

#### Crystal data

C_5_H_8_N_3_^+^·C_24_H_20_B^−^·C_20_H_18_N_6_
*M* *_r_* = 771.76
Triclinic, 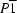

*a* = 9.2700 (8) Å
*b* = 14.5143 (10) Å
*c* = 15.9754 (13) Å
α = 93.623 (5)°
β = 104.266 (5)°
γ = 101.876 (5)°
*V* = 2023.8 (3) Å^3^
*Z* = 2
Mo *K*α radiation
μ = 0.08 mm^−1^
*T* = 150 K
0.09 × 0.07 × 0.03 mm

#### Data collection

Bruker APEXII CCD diffractometer
Absorption correction: multi-scan (*SADABS*; Bruker, 2007 ▶) *T*_min_ = 0.993, *T*_max_ = 0.998
109361 measured reflections
7416 independent reflections
4071 reflections with *I* > 2σ(*I*)
*R*_int_ = 0.177

#### Refinement

*R*[*F* ^2^ > 2σ(*F* ^2^)] = 0.061
*wR*(*F* ^2^) = 0.166
*S* = 1.00
7416 reflections
536 parameters
H-atom parameters constrained
Δρ_max_ = 1.28 e Å^−3^
Δρ_min_ = −0.25 e Å^−3^

### 

Data collection: *APEX2* (Bruker, 2007 ▶); cell refinement: *SAINT* (Bruker, 2007 ▶); data reduction: *SAINT*; program(s) used to solve structure: *SHELXS97* (Sheldrick, 2008) ▶; program(s) used to refine structure: *SHELXL97* (Sheldrick, 2008) ▶; molecular graphics: *SHELXTL* (Sheldrick, 2008) ▶; software used to prepare material for publication: *SHELXTL* ▶.

## Supplementary Material

Crystal structure: contains datablocks I, global. DOI: 10.1107/S1600536811012943/gw2100sup1.cif

Structure factors: contains datablocks I. DOI: 10.1107/S1600536811012943/gw2100Isup2.hkl

Additional supplementary materials:  crystallographic information; 3D view; checkCIF report

## Figures and Tables

**Table 1:** Hydrogen-bond geometry (Å, °)

*D*—H⋯*A*	*D*—H	H⋯*A*	*D*⋯*A*	*D*—H⋯*A*
N7—H7*A*⋯N3	0.88	2.21	3.084 (9)	177
N7—H7*B*⋯N6	0.88	2.51	3.304 (12)	150
N8—H8*A*⋯N2	0.88	2.30	3.175 (9)	177
N9—H9*A*⋯N1	0.88	2.02	2.887 (11)	170

**Fig. 1.**
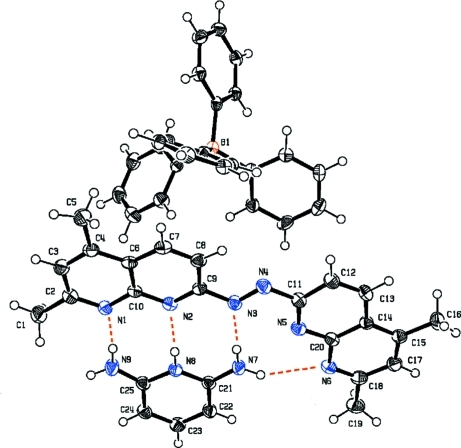
Thermal ellipsoid plot of the title compound at 50% probability level including hydrogen bonding interactions of the complex.

**Fig. 2.**
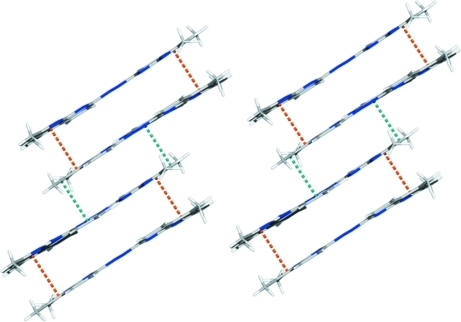
Crystal packing of the complex (borate anion is omitted for ease of visualization) showing π-π interactions.

## supplementary crystallographic information

### Comment

In the context of utility, 1,8-Naphthyridine derivatives are found to be valuable drugs and with a wide variety of pharmacological applications. They are effective fungicides and known for their antimycobacterial activity. Recent studies have revealed their ability in treatments of diabetes and related disorders. Herein, we report the crystal structure of the title compound C_49_H_46_BN_9_ that has almost coplanar naphthyridine and pyridinium moieties and an almost perfect tetrahedral borate ion. The main forces of attraction here are hydrogen bonding between the acceptor atoms, N of naphthyridine unit and the donor N—H atoms of the diaminopyridinium ion. There is also a π-π stacking interaction between adjacent parallel naphthyridyl rings. The extensions due to these interactions form the three dimensional π-stacked network structure as shown in figure 2.

Within the bis-naphthyridine molecule, the plane of the naphthyridine ring system consisting of N1 and N2 nitrogen atoms is slightly deviated from that of the second naphthyridine ring system consisting of N5 and N6 nitrogen atoms by an angle of 5.300 (4). The torsion angle between the N2, C9, N3 and N4 atoms is -176.514 (280)*°* indicative of an *anti* conformation and the torsion angle between the N5, C11, N4 and N3 is 4.165 (441)*°* indicating the *syn* conformation of the azo function with each naphthyridine ring system. The diaminopyridinium cation is complexed to the bis(1,8-naphthyridine) in an unsymmetrical fashion *via* hydrogen bonding and ion-dipole bonding. The hydrogen bonding in the complex displays head-on and bent geometries. The hydrogen bond distances are N1···N9 = 2.887 (4) Å, N2···N8 = 3.175 (4) Å, N3···N7 = 3.084 (4) Å and N6···N7 = 3.304 (4) Å with NH···N bond angles 169.989 (211)°, 177.236 (184)°, 176.497 (194)° and 150.139 (188)° respectively. The hydrogen bond distances range from 2.887 (4) Å to 3.304 (4) Å within the complex. Apart from hydrogen bonding, the naphthyridine moieties interact with the diamino pyridinium cations of adjacent complexes by π-π interactions of their terminal rings. The distance between the centroid of the N1, N2 naphthyridine ring to the plane of the N5, N6 naphthyridine ring and the distance between the centroid of the diamino pyridinium cation to C17 atom which are 3.447 (1) Å and 3.412 (3) Å respectively, strongly indicate π-π interactions. The complexes are set in a columnar arrangement with a distance of 15.531 (1) Å along *a* axis and 20.350 (1) Å along *b* axis between the centroids of the columns. The interstices of the columns are occupied by the tetraphenylborate anions. Four phenyl rings complete the slightly distorted tetrahedral geometry around each boron atom and appears non-interactive with the rest of the complex.

### Experimental

Synthesis of 1,2-bis(5,7-dimethyl-1,8-naphthyridin-2-yl)diazene: A cold solution of 2,4-dimethyl-7-amino-1,8-naphthyridine (1.02 g, 5.86 mmol) in 25 ml water was added dropwise over ten minutes to 10% sodium hypochlorite solution (36 ml, 0.58 mol). The opaque orange mixture was stirred at 0–5° C for 1 h. and extracted using 3x15 ml of dichloromethane. The organic layers were pooled and dried over anhydrous magnesuim sulfate and the solvent was reduced under vacuum to give orange crude solid. The crude product was purified by chromatography on Al_2_O_3_ (eluent: acetone/hexane 1/10) in 76% yield.

Synthesis of 2,6-Diaminopyridinium tetrakisphenylborate: The synthesis of (C_5_H_8_N_3_^+^[BPh_4_^-^]) was carried out by adding solution of sodium tetrakisphenylborate (1.65 g, 3.45 mmol, 5 eq.) in water (5 ml) to a a solution of 2,6-di aminopyridine hydrochloride (100 mg, 0.69 mmol) in water (5 ml). After stirring the solution at room temperature for 20 minutes, the resulting precipitate was filtered and washed with small aliquots of water (4x 3 mL) and dried to yield the pure title complex.

Crystallization was carried out by dissolving the 1,2-bis(5,7-dimethyl-1,8-naphthyridin-2-yl)diazene (0.0051 g 0.015 mmol) and 2,6-Diaminopyridinium tetrakisphenylborate (0.0063 g, 0.015 mmoL) in 0.5 ml of acetonitrile and allowing diisopropyl ether (0.5 ml) to slowly diffuse into the acetonitrile solution of the complex. Red plates of the complex developed overnight and were subjected to diffraction at 150 (2) K.

### Refinement

The highest residual peak and deepest hole in the final difference map were located at 0.87 Å and 0.38 Å from the H17 and H1B atoms respectively.

### Crystal data

**Table:**

C_5_H_8_N_3_^+^·C_24_H_20_B^−^·C_20_H_18_N_6_	*Z* = 2
*M**_r_* = 771.76	*F*(000) = 816
Triclinic, *P*1	*D*_x_ = 1.266 Mg m^−^^3^
Hall symbol: -P 1	Mo *K*α radiation, λ = 0.71073 Å
*a* = 9.2700 (8) Å	Cell parameters from 4472 reflections
*b* = 14.5143 (10) Å	θ = 2.3–22.3°
*c* = 15.9754 (13) Å	µ = 0.08 mm^−^^1^
α = 93.623 (5)°	*T* = 150 K
β = 104.266 (5)°	Plate, red
γ = 101.876 (5)°	0.09 × 0.07 × 0.03 mm
*V* = 2023.8 (3) Å^3^	

### Data collection

**Table:**

Bruker APEXII CCD diffractometer	7416 independent reflections
Radiation source: fine-focus sealed tube	4071 reflections with *I* > 2σ(*I*)
graphite	*R*_int_ = 0.177
φ and ω scans	θ_max_ = 25.4°, θ_min_ = 1.8°
Absorption correction: multi-scan (*SADABS*; Bruker, 2007)	*h* = −11→11
*T*_min_ = 0.993, *T*_max_ = 0.998	*k* = −17→17
109361 measured reflections	*l* = −19→19

### Refinement

**Table:**

Refinement on *F*^2^	Primary atom site location: structure-invariant direct methods
Least-squares matrix: full	Secondary atom site location: difference Fourier map
*R*[*F*^2^ > 2σ(*F*^2^)] = 0.061	Hydrogen site location: inferred from neighbouring sites
*wR*(*F*^2^) = 0.166	H-atom parameters constrained
*S* = 1.00	*w* = 1/[σ^2^(*F*_o_^2^) + (0.0614*P*)^2^ + 1.9563*P*] where *P* = (*F*_o_^2^ + 2*F*_c_^2^)/3
7416 reflections	(Δ/σ)_max_ < 0.001
536 parameters	Δρ_max_ = 1.28 e Å^−^^3^
0 restraints	Δρ_min_ = −0.25 e Å^−^^3^

### Special details

**Table:**

Geometry. All e.s.d.'s (except the e.s.d. in the dihedral angle between two l.s. planes) are estimated using the full covariance matrix. The cell e.s.d.'s are taken into account individually in the estimation of e.s.d.'s in distances, angles and torsion angles; correlations between e.s.d.'s in cell parameters are only used when they are defined by crystal symmetry. An approximate (isotropic) treatment of cell e.s.d.'s is used for estimating e.s.d.'s involving l.s. planes.
Refinement. Refinement of *F*^2^ against ALL reflections. The weighted *R*-factor *wR* and goodness of fit *S* are based on *F*^2^, conventional *R*-factors *R* are based on *F*, with *F* set to zero for negative *F*^2^. The threshold expression of *F*^2^ > σ(*F*^2^) is used only for calculating *R*-factors(gt) *etc*. and is not relevant to the choice of reflections for refinement. *R*-factors based on *F*^2^ are statistically about twice as large as those based on *F*, and *R*- factors based on ALL data will be even larger.

### Fractional atomic coordinates and isotropic or equivalent isotropic displacement parameters (Å^2^)

**Table:**

	*x*	*y*	*z*	*U*_iso_*/*U*_eq_	
C1	−0.0022 (4)	0.6545 (2)	0.4229 (2)	0.0400 (9)	
H1A	0.1078	0.6666	0.4506	0.060*	
H1B	−0.0576	0.6544	0.4679	0.060*	
H1C	−0.0232	0.7043	0.3862	0.060*	
C2	−0.0538 (4)	0.5599 (2)	0.3681 (2)	0.0304 (8)	
C3	−0.1584 (4)	0.4860 (2)	0.3901 (2)	0.0310 (8)	
H3	−0.1940	0.4960	0.4401	0.037*	
C4	−0.2084 (4)	0.4000 (2)	0.3396 (2)	0.0279 (8)	
C5	−0.3193 (4)	0.3201 (2)	0.3621 (2)	0.0379 (9)	
H5A	−0.3539	0.3432	0.4110	0.057*	
H5B	−0.2685	0.2685	0.3784	0.057*	
H5C	−0.4075	0.2967	0.3116	0.057*	
C6	−0.1513 (4)	0.3876 (2)	0.2658 (2)	0.0248 (7)	
C10	−0.0435 (4)	0.4647 (2)	0.2495 (2)	0.0239 (7)	
C8	−0.1281 (4)	0.2975 (2)	0.1409 (2)	0.0278 (8)	
H8	−0.1547	0.2411	0.1015	0.033*	
C9	−0.0201 (4)	0.3773 (2)	0.1314 (2)	0.0255 (7)	
C11	0.0958 (4)	0.2935 (2)	−0.0507 (2)	0.0273 (8)	
C7	−0.1935 (4)	0.3032 (2)	0.2082 (2)	0.0286 (8)	
H7	−0.2669	0.2506	0.2161	0.034*	
C12	0.0664 (4)	0.2038 (2)	−0.0984 (2)	0.0307 (8)	
H12	0.0038	0.1500	−0.0839	0.037*	
C13	0.1303 (4)	0.1963 (2)	−0.1658 (2)	0.0299 (8)	
H13	0.1102	0.1369	−0.2003	0.036*	
C14	0.2263 (4)	0.2763 (2)	−0.1846 (2)	0.0247 (7)	
C15	0.3010 (4)	0.2757 (2)	−0.2525 (2)	0.0289 (8)	
C16	0.2785 (4)	0.1871 (2)	−0.3127 (2)	0.0381 (9)	
H16A	0.3433	0.1997	−0.3527	0.057*	
H16B	0.1710	0.1673	−0.3462	0.057*	
H16C	0.3064	0.1366	−0.2786	0.057*	
C17	0.3947 (4)	0.3599 (2)	−0.2600 (2)	0.0314 (8)	
H17	0.4472	0.3623	−0.3042	0.038*	
C18	0.4143 (4)	0.4434 (2)	−0.2028 (2)	0.0306 (8)	
C19	0.5224 (4)	0.5332 (2)	−0.2113 (2)	0.0397 (9)	
H19A	0.4934	0.5881	−0.1868	0.059*	
H19B	0.5171	0.5377	−0.2728	0.059*	
H19C	0.6270	0.5321	−0.1797	0.059*	
C20	0.2503 (4)	0.3635 (2)	−0.1314 (2)	0.0258 (8)	
C21	0.2972 (4)	0.6386 (2)	0.0729 (2)	0.0253 (8)	
C22	0.3877 (4)	0.7207 (2)	0.0572 (2)	0.0291 (8)	
H22	0.4334	0.7199	0.0102	0.035*	
C23	0.4104 (4)	0.8035 (2)	0.1107 (2)	0.0311 (8)	
H23	0.4703	0.8602	0.0990	0.037*	
C24	0.3484 (4)	0.8065 (2)	0.1808 (2)	0.0294 (8)	
H24	0.3652	0.8644	0.2168	0.035*	
C25	0.2613 (4)	0.7237 (2)	0.1979 (2)	0.0264 (8)	
C26	0.1943 (4)	0.2941 (2)	0.3487 (2)	0.0311 (8)	
H26	0.1188	0.2950	0.2965	0.037*	
C27	0.2582 (4)	0.3782 (3)	0.4048 (2)	0.0392 (9)	
H27	0.2258	0.4346	0.3906	0.047*	
C28	0.3695 (4)	0.3794 (3)	0.4815 (2)	0.0345 (9)	
H28	0.4139	0.4363	0.5202	0.041*	
C29	0.4139 (4)	0.2964 (2)	0.5004 (2)	0.0326 (8)	
H29	0.4901	0.2962	0.5525	0.039*	
C30	0.3483 (4)	0.2130 (2)	0.4441 (2)	0.0265 (8)	
H30	0.3806	0.1569	0.4594	0.032*	
C31	0.2364 (3)	0.2082 (2)	0.3658 (2)	0.0231 (7)	
C32	0.2986 (4)	0.2010 (2)	0.1835 (2)	0.0270 (8)	
H32	0.3137	0.2593	0.2186	0.032*	
C33	0.3457 (4)	0.2021 (3)	0.1072 (2)	0.0349 (9)	
H33	0.3930	0.2606	0.0915	0.042*	
C34	0.3244 (4)	0.1191 (3)	0.0541 (2)	0.0349 (9)	
H34	0.3558	0.1200	0.0018	0.042*	
C35	0.2568 (4)	0.0346 (2)	0.0780 (2)	0.0302 (8)	
H35	0.2408	−0.0232	0.0420	0.036*	
C36	0.2124 (4)	0.0343 (2)	0.1546 (2)	0.0255 (7)	
H36	0.1677	−0.0248	0.1703	0.031*	
C37	0.2299 (3)	0.1169 (2)	0.21029 (19)	0.0212 (7)	
C38	−0.1165 (3)	0.0656 (2)	0.1803 (2)	0.0228 (7)	
H38	−0.0681	0.0573	0.1354	0.027*	
C39	−0.2754 (4)	0.0488 (2)	0.1590 (2)	0.0251 (7)	
H39	−0.3325	0.0313	0.0999	0.030*	
C40	−0.3512 (4)	0.0574 (2)	0.2226 (2)	0.0265 (8)	
H40	−0.4597	0.0445	0.2081	0.032*	
C41	−0.2647 (4)	0.0852 (2)	0.3080 (2)	0.0263 (7)	
H41	−0.3143	0.0911	0.3529	0.032*	
C42	−0.1064 (4)	0.1046 (2)	0.3282 (2)	0.0250 (7)	
H42	−0.0502	0.1257	0.3870	0.030*	
C43	−0.0252 (3)	0.0944 (2)	0.2663 (2)	0.0217 (7)	
C44	0.1203 (4)	−0.0330 (2)	0.3938 (2)	0.0255 (7)	
H44	0.0220	−0.0220	0.3918	0.031*	
C45	0.1699 (4)	−0.1019 (2)	0.4433 (2)	0.0311 (8)	
H45	0.1057	−0.1365	0.4741	0.037*	
C46	0.3121 (4)	−0.1201 (2)	0.4477 (2)	0.0294 (8)	
H46	0.3461	−0.1672	0.4811	0.035*	
C47	0.4040 (4)	−0.0684 (2)	0.4026 (2)	0.0270 (8)	
H47	0.5025	−0.0793	0.4053	0.032*	
C48	0.3519 (4)	−0.0005 (2)	0.3533 (2)	0.0236 (7)	
H48	0.4165	0.0334	0.3222	0.028*	
C49	0.2092 (3)	0.0205 (2)	0.34715 (19)	0.0213 (7)	
B1	0.1629 (4)	0.1103 (3)	0.2967 (2)	0.0220 (8)	
N1	0.0032 (3)	0.55059 (18)	0.30024 (17)	0.0270 (6)	
N2	0.0221 (3)	0.45896 (18)	0.18203 (17)	0.0258 (6)	
N3	0.0568 (3)	0.37649 (19)	0.06346 (17)	0.0267 (6)	
N4	0.0220 (3)	0.2969 (2)	0.01827 (18)	0.0293 (7)	
N5	0.1833 (3)	0.37119 (18)	−0.06553 (17)	0.0281 (7)	
N6	0.3438 (3)	0.44677 (18)	−0.14110 (18)	0.0289 (7)	
N7	0.2625 (3)	0.55485 (19)	0.02383 (17)	0.0307 (7)	
H7A	0.2029	0.5055	0.0369	0.037*	
H7B	0.2993	0.5491	−0.0215	0.037*	
N8	0.2360 (3)	0.64251 (18)	0.14261 (16)	0.0253 (6)	
H8A	0.1778	0.5904	0.1523	0.030*	
N9	0.1999 (4)	0.7172 (2)	0.26545 (18)	0.0381 (8)	
H9A	0.1460	0.6624	0.2729	0.046*	
H9B	0.2135	0.7677	0.3025	0.046*	

### Atomic displacement parameters (Å^2^)

**Table:**

	*U*^11^	*U*^22^	*U*^33^	*U*^12^	*U*^13^	*U*^23^
C1	0.051 (2)	0.031 (2)	0.040 (2)	0.0054 (18)	0.0177 (19)	0.0012 (17)
C2	0.0304 (19)	0.028 (2)	0.0332 (19)	0.0081 (16)	0.0083 (16)	0.0038 (16)
C3	0.031 (2)	0.032 (2)	0.0320 (19)	0.0080 (16)	0.0111 (16)	0.0055 (16)
C4	0.0272 (18)	0.027 (2)	0.0334 (19)	0.0086 (15)	0.0111 (15)	0.0101 (16)
C5	0.039 (2)	0.035 (2)	0.043 (2)	0.0053 (17)	0.0182 (18)	0.0080 (17)
C6	0.0225 (17)	0.0244 (19)	0.0273 (18)	0.0067 (14)	0.0036 (14)	0.0086 (15)
C10	0.0247 (17)	0.0219 (18)	0.0259 (17)	0.0074 (15)	0.0056 (15)	0.0061 (14)
C8	0.0320 (19)	0.0220 (19)	0.0246 (17)	0.0021 (15)	0.0021 (15)	0.0035 (14)
C9	0.0271 (18)	0.0265 (19)	0.0232 (17)	0.0086 (15)	0.0045 (14)	0.0057 (15)
C11	0.0248 (18)	0.026 (2)	0.0300 (18)	0.0049 (15)	0.0064 (15)	0.0014 (15)
C7	0.0282 (19)	0.0254 (19)	0.0305 (19)	0.0032 (15)	0.0059 (15)	0.0085 (15)
C12	0.0295 (19)	0.024 (2)	0.034 (2)	0.0011 (15)	0.0064 (16)	−0.0005 (15)
C13	0.0312 (19)	0.0227 (19)	0.0333 (19)	0.0046 (15)	0.0068 (16)	−0.0028 (15)
C14	0.0248 (18)	0.0250 (19)	0.0248 (17)	0.0086 (15)	0.0050 (14)	0.0034 (14)
C15	0.0292 (19)	0.030 (2)	0.0285 (18)	0.0141 (16)	0.0028 (15)	0.0055 (15)
C16	0.045 (2)	0.036 (2)	0.036 (2)	0.0103 (18)	0.0164 (18)	−0.0015 (17)
C17	0.031 (2)	0.034 (2)	0.0330 (19)	0.0117 (17)	0.0116 (16)	0.0074 (16)
C18	0.0258 (19)	0.030 (2)	0.035 (2)	0.0059 (15)	0.0059 (16)	0.0086 (16)
C19	0.039 (2)	0.032 (2)	0.049 (2)	0.0049 (18)	0.0147 (19)	0.0100 (18)
C20	0.0251 (18)	0.0220 (19)	0.0285 (18)	0.0064 (15)	0.0030 (15)	0.0020 (15)
C21	0.0256 (18)	0.0241 (19)	0.0252 (17)	0.0076 (15)	0.0035 (15)	0.0031 (15)
C22	0.0315 (19)	0.0240 (19)	0.0314 (19)	0.0030 (15)	0.0105 (16)	0.0012 (15)
C23	0.031 (2)	0.025 (2)	0.036 (2)	−0.0006 (15)	0.0103 (16)	0.0052 (16)
C24	0.0317 (19)	0.0196 (18)	0.0343 (19)	0.0021 (15)	0.0080 (16)	−0.0004 (15)
C25	0.0277 (18)	0.0224 (19)	0.0274 (18)	0.0046 (15)	0.0054 (15)	0.0017 (15)
C26	0.0285 (19)	0.032 (2)	0.0317 (19)	0.0089 (16)	0.0040 (15)	0.0028 (16)
C27	0.050 (2)	0.026 (2)	0.042 (2)	0.0108 (18)	0.0122 (19)	0.0004 (17)
C28	0.037 (2)	0.030 (2)	0.033 (2)	0.0001 (17)	0.0120 (17)	−0.0062 (16)
C29	0.0281 (19)	0.038 (2)	0.0261 (18)	0.0005 (16)	0.0054 (15)	−0.0028 (16)
C30	0.0263 (18)	0.0284 (19)	0.0260 (17)	0.0051 (15)	0.0106 (15)	0.0016 (15)
C31	0.0197 (17)	0.0272 (19)	0.0248 (17)	0.0049 (14)	0.0104 (14)	0.0041 (14)
C32	0.0280 (18)	0.0259 (19)	0.0285 (18)	0.0053 (15)	0.0115 (15)	0.0013 (15)
C33	0.040 (2)	0.035 (2)	0.033 (2)	0.0036 (17)	0.0178 (17)	0.0129 (17)
C34	0.036 (2)	0.048 (2)	0.0246 (18)	0.0108 (18)	0.0145 (16)	0.0063 (17)
C35	0.0314 (19)	0.035 (2)	0.0245 (18)	0.0095 (16)	0.0083 (15)	−0.0037 (15)
C36	0.0217 (17)	0.0265 (19)	0.0279 (18)	0.0045 (14)	0.0065 (14)	0.0037 (15)
C37	0.0134 (15)	0.0256 (18)	0.0229 (16)	0.0043 (13)	0.0020 (13)	0.0019 (14)
C38	0.0231 (17)	0.0207 (18)	0.0264 (17)	0.0055 (14)	0.0095 (14)	0.0031 (14)
C39	0.0212 (17)	0.0252 (19)	0.0257 (17)	0.0026 (14)	0.0025 (14)	0.0036 (14)
C40	0.0158 (16)	0.0262 (19)	0.0359 (19)	0.0026 (14)	0.0064 (15)	0.0029 (15)
C41	0.0235 (18)	0.0271 (19)	0.0332 (19)	0.0076 (15)	0.0145 (15)	0.0063 (15)
C42	0.0237 (18)	0.0278 (19)	0.0226 (17)	0.0057 (15)	0.0050 (14)	0.0013 (14)
C43	0.0208 (17)	0.0197 (17)	0.0248 (17)	0.0035 (14)	0.0071 (14)	0.0029 (13)
C44	0.0200 (17)	0.0273 (19)	0.0292 (18)	0.0027 (14)	0.0094 (14)	0.0019 (15)
C45	0.036 (2)	0.028 (2)	0.0286 (18)	0.0024 (16)	0.0104 (16)	0.0057 (15)
C46	0.035 (2)	0.0248 (19)	0.0266 (18)	0.0090 (16)	0.0036 (15)	0.0044 (15)
C47	0.0244 (18)	0.0285 (19)	0.0282 (18)	0.0095 (15)	0.0059 (15)	−0.0016 (15)
C48	0.0222 (17)	0.0242 (18)	0.0242 (17)	0.0030 (14)	0.0080 (14)	0.0014 (14)
C49	0.0196 (16)	0.0222 (18)	0.0191 (16)	0.0009 (14)	0.0047 (13)	−0.0037 (13)
B1	0.0169 (18)	0.026 (2)	0.0214 (18)	0.0031 (16)	0.0046 (15)	0.0003 (16)
N1	0.0303 (16)	0.0236 (16)	0.0263 (15)	0.0054 (13)	0.0069 (13)	0.0020 (12)
N2	0.0262 (15)	0.0218 (16)	0.0281 (15)	0.0050 (12)	0.0050 (12)	0.0039 (12)
N3	0.0276 (16)	0.0246 (16)	0.0255 (15)	0.0053 (13)	0.0040 (12)	0.0004 (13)
N4	0.0297 (16)	0.0255 (16)	0.0308 (16)	0.0053 (13)	0.0056 (13)	0.0016 (13)
N5	0.0281 (16)	0.0216 (16)	0.0314 (16)	0.0016 (13)	0.0058 (13)	0.0011 (12)
N6	0.0274 (16)	0.0229 (16)	0.0357 (16)	0.0048 (13)	0.0080 (13)	0.0033 (13)
N7	0.0386 (17)	0.0246 (16)	0.0268 (15)	0.0026 (13)	0.0101 (13)	−0.0020 (13)
N8	0.0268 (15)	0.0198 (15)	0.0280 (15)	0.0018 (12)	0.0080 (12)	0.0028 (12)
N9	0.054 (2)	0.0242 (16)	0.0350 (17)	−0.0028 (14)	0.0217 (15)	−0.0037 (13)

### Geometric parameters (Å, °)

**Table:**

C1—C2	1.501 (5)	C25—N8	1.370 (4)
C1—H1A	0.9800	C26—C27	1.395 (5)
C1—H1B	0.9800	C26—C31	1.405 (4)
C1—H1C	0.9800	C26—H26	0.9500
C2—N1	1.328 (4)	C27—C28	1.390 (5)
C2—C3	1.416 (5)	C27—H27	0.9500
C3—C4	1.369 (5)	C28—C29	1.379 (5)
C3—H3	0.9500	C28—H28	0.9500
C4—C6	1.422 (4)	C29—C30	1.392 (4)
C4—C5	1.508 (5)	C29—H29	0.9500
C5—H5A	0.9800	C30—C31	1.402 (4)
C5—H5B	0.9800	C30—H30	0.9500
C5—H5C	0.9800	C31—B1	1.653 (5)
C6—C7	1.409 (4)	C32—C33	1.393 (4)
C6—C10	1.422 (4)	C32—C37	1.399 (4)
C10—N2	1.368 (4)	C32—H32	0.9500
C10—N1	1.370 (4)	C33—C34	1.379 (5)
C8—C7	1.363 (5)	C33—H33	0.9500
C8—C9	1.410 (4)	C34—C35	1.379 (5)
C8—H8	0.9500	C34—H34	0.9500
C9—N2	1.321 (4)	C35—C36	1.385 (4)
C9—N3	1.439 (4)	C35—H35	0.9500
C11—N5	1.317 (4)	C36—C37	1.403 (4)
C11—C12	1.406 (4)	C36—H36	0.9500
C11—N4	1.437 (4)	C37—B1	1.648 (5)
C7—H7	0.9500	C38—C39	1.393 (4)
C12—C13	1.361 (5)	C38—C43	1.409 (4)
C12—H12	0.9500	C38—H38	0.9500
C13—C14	1.407 (5)	C39—C40	1.382 (4)
C13—H13	0.9500	C39—H39	0.9500
C14—C15	1.424 (5)	C40—C41	1.388 (4)
C14—C20	1.424 (4)	C40—H40	0.9500
C15—C17	1.377 (5)	C41—C42	1.387 (4)
C15—C16	1.503 (5)	C41—H41	0.9500
C16—H16A	0.9800	C42—C43	1.401 (4)
C16—H16B	0.9800	C42—H42	0.9500
C16—H16C	0.9800	C43—B1	1.653 (4)
C17—C18	1.423 (5)	C44—C45	1.395 (4)
C17—H17	0.9500	C44—C49	1.396 (4)
C18—N6	1.313 (4)	C44—H44	0.9500
C18—C19	1.507 (5)	C45—C46	1.383 (5)
C19—H19A	0.9800	C45—H45	0.9500
C19—H19B	0.9800	C46—C47	1.384 (5)
C19—H19C	0.9800	C46—H46	0.9500
C20—N5	1.357 (4)	C47—C48	1.389 (4)
C20—N6	1.377 (4)	C47—H47	0.9500
C21—N7	1.337 (4)	C48—C49	1.400 (4)
C21—N8	1.372 (4)	C48—H48	0.9500
C21—C22	1.385 (4)	C49—B1	1.650 (5)
C22—C23	1.377 (4)	N3—N4	1.259 (3)
C22—H22	0.9500	N7—H7A	0.8800
C23—C24	1.382 (5)	N7—H7B	0.8800
C23—H23	0.9500	N8—H8A	0.8800
C24—C25	1.386 (4)	N9—H9A	0.8800
C24—H24	0.9500	N9—H9B	0.8800
C25—N9	1.338 (4)		
			
C2—C1—H1A	109.5	C28—C27—C26	120.0 (3)
C2—C1—H1B	109.5	C28—C27—H27	120.0
H1A—C1—H1B	109.5	C26—C27—H27	120.0
C2—C1—H1C	109.5	C29—C28—C27	118.7 (3)
H1A—C1—H1C	109.5	C29—C28—H28	120.7
H1B—C1—H1C	109.5	C27—C28—H28	120.7
N1—C2—C3	123.3 (3)	C28—C29—C30	120.7 (3)
N1—C2—C1	117.0 (3)	C28—C29—H29	119.6
C3—C2—C1	119.7 (3)	C30—C29—H29	119.6
C4—C3—C2	120.2 (3)	C29—C30—C31	122.8 (3)
C4—C3—H3	119.9	C29—C30—H30	118.6
C2—C3—H3	119.9	C31—C30—H30	118.6
C3—C4—C6	118.1 (3)	C30—C31—C26	114.8 (3)
C3—C4—C5	121.0 (3)	C30—C31—B1	123.1 (3)
C6—C4—C5	120.9 (3)	C26—C31—B1	122.1 (3)
C4—C5—H5A	109.5	C33—C32—C37	122.3 (3)
C4—C5—H5B	109.5	C33—C32—H32	118.9
H5A—C5—H5B	109.5	C37—C32—H32	118.9
C4—C5—H5C	109.5	C34—C33—C32	120.5 (3)
H5A—C5—H5C	109.5	C34—C33—H33	119.7
H5B—C5—H5C	109.5	C32—C33—H33	119.7
C7—C6—C4	124.0 (3)	C33—C34—C35	119.1 (3)
C7—C6—C10	117.8 (3)	C33—C34—H34	120.4
C4—C6—C10	118.2 (3)	C35—C34—H34	120.4
N2—C10—N1	114.6 (3)	C34—C35—C36	119.8 (3)
N2—C10—C6	122.7 (3)	C34—C35—H35	120.1
N1—C10—C6	122.7 (3)	C36—C35—H35	120.1
C7—C8—C9	118.3 (3)	C35—C36—C37	123.3 (3)
C7—C8—H8	120.8	C35—C36—H36	118.3
C9—C8—H8	120.8	C37—C36—H36	118.3
N2—C9—C8	125.1 (3)	C32—C37—C36	115.0 (3)
N2—C9—N3	113.0 (3)	C32—C37—B1	125.1 (3)
C8—C9—N3	121.9 (3)	C36—C37—B1	119.8 (3)
N5—C11—C12	124.9 (3)	C39—C38—C43	122.3 (3)
N5—C11—N4	120.0 (3)	C39—C38—H38	118.9
C12—C11—N4	115.1 (3)	C43—C38—H38	118.9
C8—C7—C6	119.5 (3)	C40—C39—C38	120.9 (3)
C8—C7—H7	120.3	C40—C39—H39	119.6
C6—C7—H7	120.3	C38—C39—H39	119.6
C13—C12—C11	118.0 (3)	C39—C40—C41	118.4 (3)
C13—C12—H12	121.0	C39—C40—H40	120.8
C11—C12—H12	121.0	C41—C40—H40	120.8
C12—C13—C14	120.1 (3)	C42—C41—C40	120.3 (3)
C12—C13—H13	120.0	C42—C41—H41	119.8
C14—C13—H13	120.0	C40—C41—H41	119.8
C13—C14—C15	124.5 (3)	C41—C42—C43	123.2 (3)
C13—C14—C20	117.3 (3)	C41—C42—H42	118.4
C15—C14—C20	118.1 (3)	C43—C42—H42	118.4
C17—C15—C14	116.9 (3)	C42—C43—C38	114.9 (3)
C17—C15—C16	121.8 (3)	C42—C43—B1	120.3 (3)
C14—C15—C16	121.3 (3)	C38—C43—B1	124.7 (3)
C15—C16—H16A	109.5	C45—C44—C49	122.7 (3)
C15—C16—H16B	109.5	C45—C44—H44	118.6
H16A—C16—H16B	109.5	C49—C44—H44	118.6
C15—C16—H16C	109.5	C46—C45—C44	120.4 (3)
H16A—C16—H16C	109.5	C46—C45—H45	119.8
H16B—C16—H16C	109.5	C44—C45—H45	119.8
C15—C17—C18	121.2 (3)	C45—C46—C47	118.7 (3)
C15—C17—H17	119.4	C45—C46—H46	120.6
C18—C17—H17	119.4	C47—C46—H46	120.6
N6—C18—C17	123.3 (3)	C46—C47—C48	120.0 (3)
N6—C18—C19	117.2 (3)	C46—C47—H47	120.0
C17—C18—C19	119.4 (3)	C48—C47—H47	120.0
C18—C19—H19A	109.5	C47—C48—C49	123.3 (3)
C18—C19—H19B	109.5	C47—C48—H48	118.3
H19A—C19—H19B	109.5	C49—C48—H48	118.3
C18—C19—H19C	109.5	C44—C49—C48	114.9 (3)
H19A—C19—H19C	109.5	C44—C49—B1	124.1 (3)
H19B—C19—H19C	109.5	C48—C49—B1	120.6 (3)
N5—C20—N6	114.0 (3)	C37—B1—C49	109.5 (3)
N5—C20—C14	122.4 (3)	C37—B1—C31	111.1 (3)
N6—C20—C14	123.6 (3)	C49—B1—C31	108.1 (2)
N7—C21—N8	116.9 (3)	C37—B1—C43	109.4 (2)
N7—C21—C22	124.6 (3)	C49—B1—C43	109.6 (3)
N8—C21—C22	118.5 (3)	C31—B1—C43	109.2 (3)
C23—C22—C21	119.1 (3)	C2—N1—C10	117.5 (3)
C23—C22—H22	120.5	C9—N2—C10	116.5 (3)
C21—C22—H22	120.5	N4—N3—C9	112.6 (3)
C22—C23—C24	121.9 (3)	N3—N4—C11	114.2 (3)
C22—C23—H23	119.1	C11—N5—C20	117.3 (3)
C24—C23—H23	119.1	C18—N6—C20	116.9 (3)
C23—C24—C25	119.0 (3)	C21—N7—H7A	120.0
C23—C24—H24	120.5	C21—N7—H7B	120.0
C25—C24—H24	120.5	H7A—N7—H7B	120.0
N9—C25—N8	117.2 (3)	C25—N8—C21	123.1 (3)
N9—C25—C24	124.4 (3)	C25—N8—H8A	118.5
N8—C25—C24	118.4 (3)	C21—N8—H8A	118.5
C27—C26—C31	123.0 (3)	C25—N9—H9A	120.0
C27—C26—H26	118.5	C25—N9—H9B	120.0
C31—C26—H26	118.5	H9A—N9—H9B	120.0
			
N1—C2—C3—C4	−1.3 (5)	C41—C42—C43—B1	−175.0 (3)
C1—C2—C3—C4	178.8 (3)	C39—C38—C43—C42	0.2 (4)
C2—C3—C4—C6	0.5 (5)	C39—C38—C43—B1	176.8 (3)
C2—C3—C4—C5	179.8 (3)	C49—C44—C45—C46	−0.2 (5)
C3—C4—C6—C7	−179.7 (3)	C44—C45—C46—C47	0.3 (5)
C5—C4—C6—C7	1.0 (5)	C45—C46—C47—C48	−0.7 (5)
C3—C4—C6—C10	1.1 (4)	C46—C47—C48—C49	1.0 (5)
C5—C4—C6—C10	−178.1 (3)	C45—C44—C49—C48	0.3 (4)
C7—C6—C10—N2	−1.5 (5)	C45—C44—C49—B1	−173.1 (3)
C4—C6—C10—N2	177.7 (3)	C47—C48—C49—C44	−0.7 (4)
C7—C6—C10—N1	178.6 (3)	C47—C48—C49—B1	172.9 (3)
C4—C6—C10—N1	−2.1 (5)	C32—C37—B1—C49	−138.5 (3)
C7—C8—C9—N2	−1.2 (5)	C36—C37—B1—C49	45.1 (4)
C7—C8—C9—N3	178.6 (3)	C32—C37—B1—C31	−19.2 (4)
C9—C8—C7—C6	−0.2 (5)	C36—C37—B1—C31	164.3 (3)
C4—C6—C7—C8	−177.7 (3)	C32—C37—B1—C43	101.4 (3)
C10—C6—C7—C8	1.4 (5)	C36—C37—B1—C43	−75.1 (3)
N5—C11—C12—C13	−1.2 (5)	C44—C49—B1—C37	−148.7 (3)
N4—C11—C12—C13	178.6 (3)	C48—C49—B1—C37	38.2 (4)
C11—C12—C13—C14	1.8 (5)	C44—C49—B1—C31	90.2 (3)
C12—C13—C14—C15	178.8 (3)	C48—C49—B1—C31	−82.9 (3)
C12—C13—C14—C20	−0.9 (5)	C44—C49—B1—C43	−28.7 (4)
C13—C14—C15—C17	−178.0 (3)	C48—C49—B1—C43	158.2 (3)
C20—C14—C15—C17	1.7 (4)	C30—C31—B1—C37	−105.9 (3)
C13—C14—C15—C16	1.8 (5)	C26—C31—B1—C37	72.1 (4)
C20—C14—C15—C16	−178.5 (3)	C30—C31—B1—C49	14.3 (4)
C14—C15—C17—C18	−0.5 (5)	C26—C31—B1—C49	−167.8 (3)
C16—C15—C17—C18	179.7 (3)	C30—C31—B1—C43	133.4 (3)
C15—C17—C18—N6	−1.3 (5)	C26—C31—B1—C43	−48.6 (4)
C15—C17—C18—C19	177.8 (3)	C42—C43—B1—C37	−167.8 (3)
C13—C14—C20—N5	−0.7 (5)	C38—C43—B1—C37	15.7 (4)
C15—C14—C20—N5	179.5 (3)	C42—C43—B1—C49	72.1 (4)
C13—C14—C20—N6	178.5 (3)	C38—C43—B1—C49	−104.4 (3)
C15—C14—C20—N6	−1.2 (5)	C42—C43—B1—C31	−46.1 (4)
N7—C21—C22—C23	177.5 (3)	C38—C43—B1—C31	137.4 (3)
N8—C21—C22—C23	−1.3 (5)	C3—C2—N1—C10	0.4 (5)
C21—C22—C23—C24	1.6 (5)	C1—C2—N1—C10	−179.8 (3)
C22—C23—C24—C25	0.2 (5)	N2—C10—N1—C2	−178.5 (3)
C23—C24—C25—N9	177.6 (3)	C6—C10—N1—C2	1.4 (4)
C23—C24—C25—N8	−2.1 (5)	C8—C9—N2—C10	1.2 (5)
C31—C26—C27—C28	0.3 (5)	N3—C9—N2—C10	−178.6 (3)
C26—C27—C28—C29	−0.1 (5)	N1—C10—N2—C9	−179.9 (3)
C27—C28—C29—C30	−0.3 (5)	C6—C10—N2—C9	0.2 (4)
C28—C29—C30—C31	0.7 (5)	N2—C9—N3—N4	176.5 (3)
C29—C30—C31—C26	−0.5 (5)	C8—C9—N3—N4	−3.2 (4)
C29—C30—C31—B1	177.5 (3)	C9—N3—N4—C11	179.6 (3)
C27—C26—C31—C30	0.1 (5)	N5—C11—N4—N3	−4.2 (4)
C27—C26—C31—B1	−178.0 (3)	C12—C11—N4—N3	176.0 (3)
C37—C32—C33—C34	0.7 (5)	C12—C11—N5—C20	−0.4 (5)
C32—C33—C34—C35	−0.5 (5)	N4—C11—N5—C20	179.8 (3)
C33—C34—C35—C36	−0.3 (5)	N6—C20—N5—C11	−178.0 (3)
C34—C35—C36—C37	1.1 (5)	C14—C20—N5—C11	1.4 (5)
C33—C32—C37—C36	0.1 (5)	C17—C18—N6—C20	1.8 (5)
C33—C32—C37—B1	−176.5 (3)	C19—C18—N6—C20	−177.3 (3)
C35—C36—C37—C32	−0.9 (5)	N5—C20—N6—C18	178.8 (3)
C35—C36—C37—B1	175.9 (3)	C14—C20—N6—C18	−0.6 (5)
C43—C38—C39—C40	−1.9 (5)	N9—C25—N8—C21	−177.4 (3)
C38—C39—C40—C41	1.5 (5)	C24—C25—N8—C21	2.3 (5)
C39—C40—C41—C42	0.5 (5)	N7—C21—N8—C25	−179.5 (3)
C40—C41—C42—C43	−2.3 (5)	C22—C21—N8—C25	−0.6 (5)
C41—C42—C43—C38	1.9 (5)		

### Hydrogen-bond geometry (Å, °)

**Table:**

*D*—H···*A*	*D*—H	H···*A*	*D*···*A*	*D*—H···*A*
N7—H7A···N3	0.88	2.21	3.084 (9)	177
N7—H7B···N6	0.88	2.51	3.304 (12)	150
N8—H8A···N2	0.88	2.30	3.175 (9)	177
N9—H9A···N1	0.88	2.02	2.887 (11)	170
